# Bacterial infections in Lilongwe, Malawi: aetiology and antibiotic resistance

**DOI:** 10.1186/1471-2334-12-67

**Published:** 2012-03-21

**Authors:** Mwai H Makoka, William C Miller, Irving F Hoffman, Rushina Cholera, Peter H Gilligan, Debbie Kamwendo, Gabriel Malunga, George Joaki, Francis Martinson, Mina C Hosseinipour

**Affiliations:** 1 National AIDS Commission, Lilongwe, Malawi; 2 University of North Carolina at Chapel School of Medicine, North Carolina, USA; 3 University of North Carolina Project, Kamuzu Central Hospital, Lilongwe, Malawi

## Abstract

**Background:**

Life-threatening infections present major challenges for health systems in Malawi and the developing world because routine microbiologic culture and sensitivity testing are not performed due to lack of capacity. Use of empirical antimicrobial therapy without regular microbiologic surveillance is unable to provide adequate treatment in the face of emerging antimicrobial resistance. This study was conducted to determine antimicrobial susceptibility patterns in order to inform treatment choices and generate hospital-wide baseline data.

**Methods:**

Culture and susceptibility testing was performed on various specimens from patients presenting with possible infectious diseases at Kamuzu Central Hospital, Lilongwe, Malawi.

**Results:**

Between July 2006 and December 2007 3104 specimens from 2458 patients were evaluated, with 60.1% from the adult medical service. Common presentations were sepsis, meningitis, pneumonia and abscess. An etiologic agent was detected in 13% of patients. The most common organisms detected from blood cultures were *Staphylococcus aureus*, *Escherichia coli*, Salmonella species and *Streptococcus pneumoniae*, whereas *Streptococcus pneumoniae *and *Cryptococcus neoformans *were most frequently detected from cerebrospinal fluid. *Haemophilus influenzae *was rarely isolated. Resistance to commonly used antibiotics was observed in up to 80% of the isolates while antibiotics that were not commonly in use maintained susceptibility.

**Conclusions:**

There is widespread resistance to almost all of the antibiotics that are empirically used in Malawi. Antibiotics that have not been widely introduced in Malawi show better laboratory performance. Choices for empirical therapy in Malawi should be revised accordingly. A microbiologic surveillance system should be established and prudent use of antimicrobials promoted to improve patient care.

## Background

Life-threatening infections present major challenges for health systems in the developing world. Managing these infections requires accurate data on the prevalence of specific causative organisms and their antimicrobial susceptibility profiles. However, microbiologic surveillance data providing this crucial information are scarce. In Malawi, a typical sub-Saharan African country, routine microbiologic culture and sensitivity testing are not performed due to lack of personnel, equipment and financial resources [1]. Instead, antimicrobial therapy is empirical and a small repertoire of antimicrobials is overused. This approach, although relatively inexpensive, leads to the emergence of antibiotic resistance and hence sub-optimal clinical outcomes. Antimicrobial resistance in Malawi may also be occasioned by uncontrolled medical and veterinary use of antibiotics by the private sector and the community.

Antimicrobial resistance, an emerging global public health threat, is increasing worldwide and has been reported in Malawi [2-5]. Of particular concern is the reported resistance to many of the first-line antibiotics for treating bacteraemia and other life-threatening infections [6]. However, some of these initial studies are now outdated, had inadequate sample sizes, or were retrospective. Consequently, the problem of antimicrobial resistance requires a more complete description [1,6-8].

At Kamuzu Central Hospital (KCH), Lilongwe, Malawi we prospectively studied patients suspected of having an infection in order to identify their causative organism and the antimicrobial susceptibility using disk diffusion method. This study was conducted to inform empirical treatment choices and also to generate hospital-wide baseline data.

## Methods

## Patients and study site

We conducted a cross-sectional study at KCH, a 750-bed government medical centre serving Lilongwe district (population 1,897,167) that also provides referral services to the central region (population 5,491,034) [9]. The study period, from July 2006 to December 2007, covered two dry seasons and one and a half rainy seasons. During evaluation for admission, patients presenting with a suspected infection were evaluated using appropriate microbiologic procedures. Specimens were only collected at admission, and were not collected from those seen on out-patient basis. This report includes specimens from blood, cerebrospinal fluid (CSF), joint fluid, bone biopsy, or pus collections.

We enrolled patients with pneumonia, meningitis, bone and joint infections, peripartum infections and other febrile clinical presentations (Table 1). Patients who were already on antibiotics ^1^, too sick ^2 ^or unwilling to undergo study procedures were excluded from the study. Due to logistical constraints, patients presenting at night and on weekends were also excluded. All laboratory analysis was conducted in the University of North Carolina (UNC) Project laboratory at KCH. Institutional Review Boards in both Malawi and at UNC-Chapel Hill approved the study and considered the collection of biological samples for diagnostic purposes to be the standard of care.

**Table 1:** Characteristics of Study Population (N = 2056)

Service	Syndrome	Total N (%)*	Male N(%)	Female N(%)	Age Mean ± SD
Overall		2056	848 (43.3)	1110 (56.7)	26.0 ± 16.7

Paediatrics		543	254	218	4.4 ± 4.9

	Febrile child	391 (89.5)	192 (90.6)	166 (87.8)	

	Neonates	46 (10.5)	20 (9.4)	23 (12.2)	

Medicine		1213	506	692	34.8 ± 12.0

(adults)	Meningitis	294 (25.1)	133 (27.4)	157 (23.3)	

	Pneumonia	360 (30.7)	116 (23.9)	240 (35.7)	

	Fever/sepsis	518 (44.2)	236 (48.7)	276 (41.0)	

Obs/Gyn		127	0	127	24.7 ± 6.2

	Meningitis	9 (7.5)	0	9 (7.5)	

	Pneumonia	7 (5.8)	0	7 (5.8)	

	Fever/sepsis	14 (11.7)	0	14 (11.7)	

	Prepartum sepsis	8 (6.7)	0	8 (6.7)	

	Puerperal sepsis	46 (38.3)	0	46 (38.3)	

	Gynaecological sepsis	36 (30.0)	0	36 (30.0)	

Surgery		166	89	73	21.6 ± 15.8

(adults and children)	Osteomyelitis	9 (5.7)	3 (3.6)	5 (7.2)	

	Necrotising fasciitis	3 (1.9)	2 (2.4)	1 (1.5)	

	Abdominal Abscess	84 (53.5)	42 (50.6)	40 (58.0)	

	Burn	8 (5.1)	3 (3.6)	5 (7.2)	

	Septic arthritis	53 (33.8)	33 (39.8)	18 (26.1)	

*Categories may not sum to the total because of missing data

Prior to study implementation, information and training sessions were held with the staff of Medicine, Pediatrics, Surgery, and Obstetrics and Gynecology departments. Content of the training sessions included selection criteria for patient identification and appropriate sample collection techniques. Interpretation and use of study results were discussed at clinical meetings.

In each department, one lead staff member was identified to facilitate study implementation, including assurance that all eligible patients were identified. A study assistant was engaged full-time to track specimens from around the hospital to the laboratory, return laboratory results to the clinicians, manage study supplies and provide general logistical support to the study. We conducted monthly review meetings with the lead staff members to discuss study progress and address logistical problems.

## Data collection

We collected patient information on case record forms, standardised for each department. Clinical information included demographics, symptoms experienced in the previous week, antibiotic use in the previous three months, findings on physical examination, and clinical diagnosis. Laboratory information included specimen(s) collected, laboratory diagnosis, organisms identified and antibiotic susceptibility results.

## Laboratory methods

Specimens were collected on the clinical wards and transported directly to the UNC Project Laboratory on the grounds of KCH. The laboratory participated in external quality assurance scheme provided by the College of American Pathologists. In general, only one blood culture was collected per patient because of funding limitations. Specimens were processed within 2 hours of collection.

We used sheep blood, chocolate, malt and Mueller-Hinton agar plates from commercial sources. MacConkey and mannitol salt agars were prepared in-house from purchased dehydrated constituents. We did not perform anaerobic cultures.

## Blood cultures

The venipuncture site was disinfected with 70% alcohol and 2% tincture of iodine before collecting approximately 10 ml of blood in adults and 3 ml of blood in paediatric patients. Blood specimens were inoculated into BBL™ SEPTI-CHEK™, with slides containing chocolate, MacConkey and Malt agars. The slides, too, were inoculated and the set was incubated at 35 - 37°C in 5% CO_2_. The bottles were inverted after 4 to 6 hours and were examined daily for signs of growth. Cultures with visible growth were assessed with gram stain. We used the gram stain results to guide subcultures and susceptibility testing by disc diffusion. Specimens with growth were subcultured onto sheep blood agar plates. Specimens with no growth after incubation for seven days were declared 'no growth'. All plates were incubated in conditions similar to the bottles.

## Cerebrospinal fluid (CSF)

The site was prepared as for venipuncture. Gram and India ink stains were performed on CSF specimens. Sheep blood and chocolate agars were also inoculated and incubated at 35 - 37°C in 5% CO_2 _and were examined daily for seven days.

## Biopsiesand swabs

Biopsies and/or swabs were obtained from bone, skin and soft tissues. Gram stains were performed on all biopsies and swabs. These specimens were cultured on sheep blood, MacConkey and chocolate agars, incubated at 35 - 37°C in 5% CO_2 _and examined daily for seven days.

## Processing of isolates

Isolates were identified and processed according to standard techniques (Table 2, 3) [10]. Antibiotic susceptibilities were determined by disc diffusion on Mueller-Hinton agar, interpreted according to the CLSI guidelines and quality-controlled using *Escherichia coli *ATCC 25922 and *Staphylococcus aureus *ATCC 25923 [11]. For *Streptococcus pneumoniae*, Mueller-Hinton agar with 5% sheep blood was used including an oxacillin screening test to determine reduced susceptibility to *S. pneumoniae *[11]. Minimum inhibitory concentrations were not performed.

**Table 2:** Laboratory methods for identification and susceptibility testing

Organism	Media	Tests for presumptive identification	Susceptibility panel	Method
*Salmonellae*	BAP	Gram stain;	GNP	Disc diffusion
	MAC	TSI;		
		Indole;		
		Colony morphology;		
		Urease		

Enteric gram negative bacilli	BAP	Gram stain;	GNP	Disc diffusion
	MAC	Colony morphology		
		Indole;		
		Oxidase;		
		TSI;		
		Citrate;		
		Urease		

*N. meningitidis*	BAP	Gram stain	None	None
	CHOC	Oxidase		
	TM	Thayer-Martin		

*S. pneumoniae*	BAP	Gram stain	GPP	Disc diffusion
	CHOC	Hemolysis Catalase	Oxacillin	
		Bile solubility	screening test	

*S. aureus*	BAP	Gram stain	GPP	Disc diffusion
	CHOC	Catalase	Oxacillin	
	MSA	Coagulase		
		Staphlex		

*C. neoformans*	Sabouraud's	Gram stain	None	None
	BAP	India Ink		
		Urease		

*Haemophilus*	BAP	Gram stain	None	None
	CHOC	Oxidase		
		API-NH		

*S. pyogenes*	BAP	Haemolysis	GPP	Disc diffusion
		Gram stain		

BAP = blood agar plate, MAC = MacConkey agar, CHOC = chocolate agar, TSI = triple-sugar with iron media, TM = Thayer-Martin agar, MSA = mannitol salt agar. Coagulase = tube coagulate test using human plasma; GPP = gram positive antibiotic panel, GNP = gram negative antibiotic panel, see Table 3.

**Table 3:** Antibiotic resistant bacteria isolated from all specimens

	Gram positive organisms		*S. aureus*	*S. pneumoniae*
Antibiotic	Resistant n/N (%)	Resistant + Intermediate n/N (%)	Resistant n/N (%)	Resistant n/N (%)
Chloramphenicol	59/171 (34.5)	77/171 (45.0)	30/95 (31.6)	17/51 (33)
Clindamycin	19/188 (10.1)	72/188 (38.3)	14/143 (9.8)^†^	3/30 (10.0) ^†^
Erythromycin	62/220 (28.2)	112/220 (50.9)	51/122 (41.8)	1/67 (1.5)
Gentamicin	60/183 (32.8)	77/183 (42.1)	28/141 (19.9)	23/27 (85.2)
Oxacillin	70/230 (30.4)	84/230 (36.5)	46/147 (31.3)	11/59 (18.6)*
Tetracycline	135/222 (60.8)	150/222 (67.6)	81/129 (62.8)	32/63 (50.8)
Tmp-Sxt	149/199 (74.9)	158/199 (79.4)	88/127 (69.3)	42/47 (89.4)
	**Gram negative organisms**		***Salmonella spp*.**	
	Resistant n/N (%)	Resistant + Intermediate n/N (%)	Resistant n/N (%)	Resistant + Intermediate n/N (%)
Chloramphenicol	49/69 (71.0)	52/69 (75.4)	12/17 (70.1)	12/17 (70.6)
Gentamicin	27/80 (33.8)	31/80 (38.8)	0/19 (0.0)	2/19 (10.5)
Tmp-Sxt	53/65 (81.5)	54/65 (83.1)	12/15 (80.0)	12/15 (80.0)
Ampicillin	65/84 (77.4)	69/84 (82.1)	18/21 (85.7)	18/21 (85.7)
Ceftriaxone	16/85 (18.8)	29/85 (34.1)	0/20 (5.0)	1/20 (5.0)
Ciprofloxacin	9/75 (12.0)	20/75 (26.7)	0/22 (0.0)	0/22 (0.0)
Nalidixic acid	26/79 (32.9)	44/79 (55.7)	0/20 (0.0)	9/20 (45.0)
				

## Contaminants

Coagulase negative staphylococci, *Bacillus *spp and dipthreoids were considered contaminants when recovered from any specimen.

## Malaria testing

Malaria testing using thick and thin blood films was conducted on a stat basis in a mini-laboratory located in the consultations area. However, results for this test were not collated with the laboratory analyses described above.

## Data analyses

All data were entered into a Microsoft Office Access 2003 database. Statistical analyses were performed using Stata, version 10.0. The data were described using univariable frequencies, distributions and measures of central tendency. We used Fisher's exact test or Pearson's chi-square test for bivariable comparisons of categorical data.

## Ethical considerations

The protocol for this study was approved by Malawi's National Health Sciences Research Committee and the Institutional Review Board of the University of North Carolina at Chapel Hill School of Medicine. Informed consent was not sought because the procedures that were involved in this study were deemed to constitute standard patient care. The results from the study were used to inform patient care.

## Results

### Characteristics of the study population

During the study period, 2236 specimens were submitted from 2056 patients. Most were seen on the adult medical service (59.2%, Table 1). The population included 848 males and 1110 females. The mean age was 34.8 years for adult medical patients, 4.4 for pediatric medical patients, 21.6 for all surgery patients, and 24.7 for obstetrics/gynecology patients. Common syndromes included sepsis, meningitis, pneumonia, and among surgery patients, abscess.

### Blood culture yield

The yield of blood cultures in our patient population was high at 12.7% (Table 4). The proportion of blood cultures yielding skin contaminants was 9.2%. Among those with positive blood cultures, the organisms identified differed between the pediatric and medical services (p ≤ 0.0001). The most common organisms recovered from blood cultures of children were *S. aureus *(48%), gram negative bacilli including *E. coli *and *Salmonella *species (26%) and *S. pneumoniae *(17%). *Salmonella *isolates were not further distinguished into Typhi and non-Typhi *Salmonella*. In contrast, among adults on the medical ward *S. pneumoniae *(33%), gram negative bacilli (27%) and *S. aureus *(23%) were most commonly isolated. *Haemophilus influenzae *was isolated once from a child.

**Table 4:** Recovery of pathogens from clinical specimens^†^

Specimen type	Positive (%)	No growth (%)	Contaminated (%)	Total
Blood	241 (12.7)	1479 (78.1)	174 (9.2)	1894

Cerebrospinal fluid	23 (15.2)	128 (84.8)	0	151

Joint fluid	13 (21.7)	47 (78.3)	0	60

Pus	86 (72.3)	30 (25.2)	3 (2.5)*	119

Bone	7 (58.3)	5 (41.7)	0	12

^†^Repeat specimens were not done, and when an isolate was recovered from different specimens they were all counted. * Includes coagulase negative staphylococci and normal flora

### Other specimens

*S. aureus *was also most frequently isolated from abscesses, bones and joints (Table 5). From cerebrospinal fluid *S. pneumoniae *and *C. neoformans *were the predominant pathogens (Table 5).

**Table 5:** Organisms recovered from different specimens

Organism	Blood n (%)	CSF n (%)	Joint n (%)	Bone n (%)	Pus n (%)	Total n (%)
*S. aureus*	92 (39.0)	0	5 (38.5)	3 (42.9)	54 (62.8)	154 (42.0)
*S. pneumoniae*	56 (23.5)	10 (43.5)	3 (23.1)	0	1 (1.2)	70 (19.1)
*Streptococcus *spp.	20 (8.4)	1 (4.4)	2 (15.4)	2 (28.6)	11 (12.8)	36 (9.8)
*Salmonella *spp	20 (8.4)	1 (4.4)	3 (23.1)	0	0	24 (6.5)
Gram negative bacilli*	43 (18.5)	2 (8.7)	0	2 (28.6)	20 (23.2)	68 (18.5)
*N. meningitidis*	2 (0.8)	0	0	0	0	2 (0.5)
*C. neoformans*	2 (0.8)	9 (39.1)	0	0	0	11 (3.0)
Yeast/Candida	2 (0.8)	0	0	0	0	2 (0.5)
Total	238 (100)	23 (100)	13 (100)	7 (100)	86 (100)	367 (100)

* includes *E. coli, Klebsiella, Proteus, Pseudomonas, Citrobacter, Yersinia *and *Acinetobacter*

### Antibiotic resistance

Over one-third of gram positive bacteria were resistant to the commonly used antibiotics (chloramphenicol, ampicillin, tetracycline, trimethoprim/sulfamethoxazole, gentamicin, and erythromycin) while for gram negative this proportion was higher (Table 3). Clindamycin gave best results among the antimicrobials that were tested, against *S. aureus *in particular and also comparably against gram positive bacteria, while ceftriaxone and ciprofloxacin gave best results against the gram negatives. *S. pneumoniae *was highly resistant to cotrimoxazole, but remained susceptible to erythromycin, while *Salmonella *was resistant to chloramphenicol, ampicillin and trimethoprim/sulfamethoxazole but susceptible to ceftriaxone (Table 3).

* Isolates with oxacillin zone size of < 20 mm were considered penicillin non-susceptible (some of which would have shown susceptibility under MIC testing)

^†^Isolates displaying resistance induced by erythromycin, i.e., positive D-test, were considered clindamycin resistant.

Tmp-Sxt = co-trimoxazole.

## Discussion and Conclusions

We conducted a large, cross-sectional microbiologic study of patients with presumed infections seen at a large medical centre in Malawi. We observed that the common bacterial causes of bacteraemia differ between children and adults, and that *S. aureus *is extremely common in blood, abscesses, joints, and bone. We also observed widespread antimicrobial resistance to commonly used antibiotics.

Our findings highlight the variation in bacteremia etiology, both geographically and temporally, within sub-Saharan Africa [8,12,13]. In our setting, gram positive bacteria, especially *S. aureus *and *S. pneumoniae*, were the most common cause of bacteraemia and infections of other anatomic sites. An earlier study in neonates in Malawi observed a predominance of *Salmonella *and other gram negative bacilli in patients with bacteremia [12]. We also isolated *S. aureus *more commonly than was observed elsewhere [7,14]. However, in The Gambia, *S. aureus *and *S. pneumoniae *caused almost three quarters of bacteraemia cases in an urban hospital [15]. In addition, this is a population which did not receive the pneumococcal conjugate vaccine.

Our observation that *S. aureus *was the most commonly isolated organism must be interpreted cautiously. Robust organisms, such as *S. aureus *and *Salmonella*, are more likely to be isolated than fastidious ones [16]. Thus, the prevalence of the robust organisms could possibly be biased upwards. However, we frequently isolated *S. pneumoniae*, a less robust organism, which suggests that our aerobic culture techniques were adequate.

Although we had relatively few CSF cultures, the results were highly informative. *C. neoformans *was a common cause of meningitis, second only to *S. pneumoniae*. The high prevalence of *Cryptococcus *was most likely due to a high prevalence of HIV infection in the patient population at KCH, where up to three-quarters of medical inpatients are HIV infected. We can not comment on the frequency of tuberculous meningitis since culture for that organism was not attempted in this study population. We also observed very few cases of *Haemophilus influenzae *meningitis or bacteraemia, possibly due to the introduction of routine childhood *H. influenzae *b vaccination in 2002. *Neisseria meningitidis *also was not recovered reflecting this as a rare cause of meningitis in this population.

We found widespread bacterial resistance to almost all of our commonly used antibiotics. Our findings contrast starkly with the limited resistance observed in Malawi between 1996 and 2001, although resistance to first line antibiotics for bacteremia was observed over a decade ago [6,12]. Of particular concern is high incidence of bacteraemia caused by non-Typhi *Salmonella*, which has increased with development of multi-drug resistance [17]. In our setting, *Salmonella *is a common gram negative bacterium infecting blood and joints and it was susceptible to gentamicin and ceftriaxone in vitro. The problem of fluoroquinolone resistance in *Salmonella enterica *serotypes other than Typhi is not well studied in Africa.

In a separate Malawi study, no fluoroquinolone resistance was reported among *Salmonella *isolates [17]. In a study from Senegal, approximately 5% of isolates showed low level fluoroquinolone resistance [18]. We found none of the 25 *Salmonella *isolates tested to be nalixidic acid resistant although interestingly close to half (11/25) were nalidixic acid intermediate (Table 3). A previous study suggests *Salmonella *strains with intermediate susceptibility by disk diffusion may have MICs in a range consistent with low level ciprofloxacin resistance [19]. Unfortunately ciprofloxacin MICs were not available to determine if these nalidixic acid intermediate isolates did, in fact, have low level ciprofloxacin resistance. This issue deserves close scrutiny in the coming years.

Our findings of widespread antimicrobial resistance have a strong bearing on the current treatment choices. Malawi, like most resource poor countries, does not provide routine microbiologic culture and sensitivity testing [3]. Instead, the Malawi National Treatment Guidelines provides for initiation of antimicrobial therapy empirically, based on clinical observation and history, which is often broad spectrum [16,20-23]. However, the development of antimicrobial resistance in this context can have major clinical consequences. In Tanzania, antimicrobial resistance was a significant risk for death in children with bacteremia [23]. Antimicrobial resistance can also lead to treatment failures, prolonged drug treatment courses and protracted hospital admissions [23].

Considering the potential consequences of increased antimicrobial resistance, our findings support a review of the current antibiotic choices in Malawi. Of the antibiotics we examined, only three are not widely used and show good susceptibility. Ceftriaxone and ciprofloxacin retain activity against gram negative bacteria, including *Salmonella *spp. Gram positive organisms, including *S. aureus *and *S. pneumoniae*, remain susceptible to clindamycin, and may also be susceptible to third-generation cephalosporins. Revision of the national treatment guidelines with inclusion of clindamycin, and ciprofloxacin or ceftriaxone, coupled with promotion of their prudent use, should improve patient outcomes and potentially save costs of providing clinical services.

This study represents an account of community-acquired infections in that it included only specimens collected at admission, and did not include subsequent specimens and specimens from patients who had already been on antimicrobial therapy prior to presentation. Unfortunately, we were unable to include patients who presented to the hospital outside of the daytime hours, due to the constraints of study personnel. In addition, the maternity ward was located outside the main hospital campus, limiting the enrolment of patients there. Thus, our study population was not a complete assessment of all infections presenting to KCH during the study period. Also clinical outcomes were not collected to triangulate our laboratory findings. More advanced microbiologic testing such as minimum inhibitory concentration (MIC) and extended spectrum beta-lactamases (ESBLs), which would have helped refine some of our basic findings, were not performed. Nonetheless, we were able to enrol a large number of patients with a diverse spectrum of clinical syndromes and also perform basic identification and antimicrobial susceptibility testing. Consequently, our results provide a large microbiologic assessment of bacterial infections in Malawi.

The widespread antimicrobial resistance in our study is concerning. Choices for empirical therapy in Malawi should be revised accordingly. Newer antibiotics, though more costly, must be used more widely, but judiciously, to limit the further spread of antimicrobial resistance. The establishment of a dependable, accurate and timely microbiological service and surveillance system along with optimal use of this service by the clinicians should considerably improve the care of patients. Finally, prudent use of antimicrobials should be promoted.

## Notes

^1 ^As a referral hospital, some patients seen at KCH were already on antibiotics.

^2 ^Due to lack of routine diagnostic culture, especially blood cultures, patient perception to collection of these specimens are not always favourable, and untoward outcome in a patient who was already critically ill may be attributed to the specimen collection procedure. The effect of this exclusion has not been quantified but may be minimal.

## Competing interests

The authors declare that they have no competing interests. The study was partially funded by University of North Carolina at Chapel Hill Center for AIDS Research (CFAR) NIH funded program #P30 AI50410.

## Authors' contributions

MHM was principal investigator, wrote first drafts of the study protocol and manuscript, contributed to laboratory and data analysis and interpretation. WCM performed the statistical analysis. IFH, PHG and MCH contributed to study conception and design, data interpretation, revising manuscript for intellectual content. GM and RC participated in data entry, cleaning and analysis. GJ, FM and DK participated in study concept and design. All authors read and approved the final manuscript.

## Pre-publication history

The pre-publication history for this paper can be accessed here:

http://www.biomedcentral.com/1471-2334/12/67/prepub
